# Thinking About the Future of Cognitive Remediation Therapy Revisited: What Is Left to Solve Before Patients Have Access?

**DOI:** 10.1093/schbul/sbae075

**Published:** 2024-05-23

**Authors:** Til Wykes, Christopher R Bowie, Matteo Cella

**Affiliations:** Institute of Psychology, Psychiatry and Neuroscience, King’s College London, London, UK; South London and Maudsley NHS Foundation Trust, London, UK; Department of Psychology, Queens University, Kingston, Canada; Institute of Psychology, Psychiatry and Neuroscience, King’s College London, London, UK; South London and Maudsley NHS Foundation Trust, London, UK

**Keywords:** cognitive remediation, implementation, psychological treatment, model

## Abstract

In our previous paper on the Future of Cognitive Remediation published more than 10 years ago, we envisaged an imminent and wide implementation of cognitive remediation therapies into mental health services. This optimism was misplaced. Despite evidence of the benefits, costs, and savings of this intervention, access is still sparse. The therapy has made its way into some treatment guidance, but these documents weight the same evidence very differently, causing confusion, and do not consider barriers to implementation. This paper revisits our previous agenda and describes how some challenges were overcome but some remain. The scientific community, with its commitment to Open Science, has produced promising sets of empirical data to explore the mechanisms of treatment action. This same community needs to understand the specific and nonspecific effects of cognitive remediation if we are to provide a formulation-based approach that can be widely implemented. In the last 10 years we have learned that cognitive remediation is not “brain training” but is a holistic therapy that involves an active therapist providing motivation support, and who helps to mitigate the impact of cognitive difficulties through metacognition to develop awareness of cognitive approaches to problems. We conclude that, of course, more research is needed but, in addition and perhaps more importantly at this stage, we need more public and health professionals’ understanding of the benefits of this therapy to inform and include this approach as part of treatment regimens.

In 2011 a paper was published in this journal on issues we need to consider if cognitive remediation was going to be accepted into mental health services. It had the imposing title of “Thinking about the Future of Cognitive Remediation.”^1^ The authors, Til Wykes and Will Spaulding, roamed around the field justifying cognition as a potential target for treatment, trying to clarify potential treatment mechanisms, and suggesting how future clinical trials should be designed and reported. Some of the suggestions were picked up and some justified in the following years, but still cognitive remediation treatments are not generally available in mental health services. This is despite a highly cited meta-analysis in the same year showing the benefits of this therapy.^2^ Treatment and service gaps remain, and this paper is a reconsideration of the potential of this intervention and identifies barriers that prevent its large-scale rollout. Some knowledge gaps, such as in treatment mechanisms, may not be very important for treatment implementation but would offer an understanding of how to improve outcomes both in their effects on an individual and to different groups and services. We will try to distinguish between these options. Not all our suggestions have a firm evidence base as this reexamination will identify potential routes to treatment access that will need further empirical support.

## A Little History

Treatment development is not linear. It has stops and starts and a meandering path. The Rand Europe group reported a gap of about 19 years between a basic understanding of the problem and treatment development and then treatment being widely available.^3^ This has not been the case for cognitive remediation. Kraepelin and Bleuler both include cognition in their description of someone with a diagnosis of dementia praecox or schizophrenia as early as 1893 as a core of the disorder. These cognitive difficulties were again noted in the 1960s as problematic,^4^ so more than 60 years later we have yet to have general treatment availability. In the intervening time we have identified that cognitive difficulties are present before illness onset, remain stable after remission, and are not caused by medication.^5^ Importantly we have established a link between cognition and functional outcomes.^6^ The barrier to treatment development seems to have been the idea that cognitive difficulties were vulnerability factors for psychosis with the assumption that these were stable and therefore could not be changed. This dampened enthusiasm for any treatment, especially as there is evidence that cognitive difficulties arise before the first onset and may continue and get even worse over the course of the disorder.^7,8^ Small laboratory-based studies also found little evidence for success in changing cognitive skills. This negative evidence supported both treatment pessimism and a compensatory treatment approach that reduced cognitive load rather than improving cognition.^9^ Even if we assume that cognitive remediation began in the late 1990s when the first prototypes were tested, it is still 25 years with little treatment implementation. The previous Futures paper suggested imminent implementation as it was a “rapidly developing treatment approach.” So, what prevented this translation? An investigation of the remaining barriers is therefore timely.

## Optimism or Pessimism About Treatment Effects?

It is not disputed that cognitive difficulties in the context of a diagnosis of schizophrenia lead to poor outcomes and that rehabilitation to improve recovery can produce failure or limited effects in the presence of cognitive difficulties.^10–12^ We also now know that adding cognitive remediation to formal rehabilitation programs will improve their outcomes^13^ and prevent failure to achieve the preferred outcome.^14^ and a recent meta-analysis demonstrated benefits for cognitive remediation as an additional treatment.^15^ So, we now know that cognition is malleable, and treatment provides benefits to cognition and functioning that can be improved further if supported by additional rehabilitation. The evidence has built since a large meta-analysis was published^2^ and further tested over the following years.^16–19^ This optimism removed one barrier, but it does not explain why, when we know this therapy is beneficial and acceptable,^20^ that it is rarely available in clinical services except in a few select locations. Treatment guidance increasingly *mentions* this treatment^21–24^ and umbrella reviews of psychosocial treatments^25^ also suggest *adding* this treatment to services, but it has not emerged as a Tier 1 service in spite of the evidence. Different guidelines approach the favorable evidence differently with NICE and APA showing the least enthusiasm. However, even when the evidence is given the highest rating by healthcare decision-making bodies and funders, there seem to be other barriers to implementation in everyday clinical practice.

A consideration for all services is their cost including a therapist and other resources like access to digital tools and reliable internet. If services choose to provide cognitive remediation it is also likely to be at the expense of other services unless the intervention either reduces costs or is better than another treatment. These considerations have not been as well researched but there is evidence that cognitive remediation can reduce inpatient stays,^26^ health care costs,^27–29^ and lower medical costs when provided with supported employment.^30^ Cognitive remediation may also increase the chances that individuals will attend other rehabilitation programs,^31^ supporting longer-term and generalizable effects.

With this accumulating evidence why did implementation not pick up the pace that was previously envisaged. One reason was the continued skepticism about “brain training.” This issue became sensationalized when 70 neuropsychologists published an open letter in 2014^32^ saying that brain games do not provide a scientifically grounded way to improve cognitive functioning, with their focus not on remediating impairment, but training for the general public. This was then followed by 111 scientists saying the opposite.^33^ This controversy continued with a review paper^34^ describing a lack of supportive evidence, followed by a further paper saying the opposite.^35^ Heightening this controversy has been the number of occasions when the Federal Trade Commission has found companies making false claims for their brain training products^36,37^ and recent reviews on outcomes.^38^ This introduced public uncertainty about the potential for any approach to improve cognition. Despite this debate being about the efficacy of brain training to prevent cognitive decline or enhance functioning in healthy individuals, these commercial products have been conflated with cognitive remediation. Consequently, concerns have leaked their way into the healthcare community and have colored the view of the merits of introducing cognitive remediation into services. Cognitive remediation is different from “brain training” but in the public’s mind these approaches appear as part of the same family.

The Futures paper identified several potential issues for future research: treatment mechanism, treatment description, and potential moderators of treatment success. Knowledge about some, but not all, has been advanced since the previous publication. In this revisit, we have considered the main advances first and so lead to where gaps need to be filled and suggest how further progress will be made.

### We Need a Good Definition

*Any treatment can be disseminated only to the degree that it can be defined and distinguished from other treatments.* ^1^

What level of detail is required for a definition of a treatment? Cognitive Behavior Therapies for Psychosis (CBTp), despite having a single descriptor, were not the same as they differed in the amount of time spent on the B and the C and this was related to the outcomes.^39^ However, this has not prevented CBTp being included in treatment guidance, and in some it is also a mandated treatment.^22^ In comparison cognitive remediation treatments all had different labels thus confusing treatment regulators. Although some authors have tried to provide more specific descriptions.^40,41^ It is helpful to identify the ingredients of a successful treatment and the limits of a credible cognitive remediation therapy. The Cognitive Remediation Experts Workshop (CREW), founded more than 15 years ago, consists of principal investigators of studies of cognitive remediation and in 2012 produced an acceptable definition that encompassed the available treatments.

*Cognitive remediation is an intervention targeting cognitive deficit using scientific principles of learning with the ultimate goal of improving functional outcomes. Its effectiveness is enhanced when provided in a context (formal or informal) that provides support and opportunity for extending everyday functioning.* Cognitive Remediation Experts Workshop 2012

The definition did not change until 2023 when it was agreed to drop the words “cognitive deficit.” This decision was based on several factors. Firstly, many studies included in meta-analyses of CR treatment did not have an inclusion criterion of “cognitive deficit” and, even when they did, the definitions were different. In those same meta-analyses there is evidence that cognitive remediation for those with poorer cognition was more beneficial, showed no effect or the opposite for both cognitive and functional outcomes.^2,18^ Although many people who had poor functioning at the beginning of a study realized more benefit and retained the benefit for longer, eg, for work functioning, this effect was related to poorer functioning at the outset not on poor cognition.^42^ The definition of impairment differed with some relying on premorbid IQ and others on specific test scores. Either approach could give misleading information as it assumes that only cognitive scores in the impaired region (eg, 2 standard deviations below the average) indicate impairment. Individuals with cognitive scores within the average range can have either islets of problems or were expected to have higher scores based on their premorbid cognitive levels or their parents and siblings achievements.^43^ There is just as much variation in the empirical data, with some studies suggesting higher performance on reasoning and problem-solving predicts better outcomes^44^ and others finding no effect.^45^ Cognitive remediation can also strengthen cognitive processes that do not score in a deficit range, and this may be helpful for functioning. A few studies have tested generic vs specific training for problematic cognitive domains and have found no difference in outcome.^46,47^ Only focusing on impaired cognitive domains treatment may not provide enough positive feedback and therefore increase feelings of poor self-worth and this conjecture is supported by a user-led study showing reduced self-esteem when early improvement in cognitive remediation was not noticed.^48^ Generic remediation has the potential of providing support in poorer performing cognitive domains while also strengthening those less impaired and has the benefit of providing experiences of success. These further considerations have produced the following new cognitive remediation definition:

Cognitive remediation is an intervention targeting cognition* using scientific principles of learning with the goal of improving functional outcomes. Its effectiveness is enhanced when provided in a context (formal or informal) that provides support and opportunity for extending everyday functioning. Cognitive Remediation Experts Workshop 2023

*Attention, memory, executive function, social cognition, or metacognition

The definition also recognizes that cognitive remediation is aimed at functioning, particularly the personal goals of individuals. This emphasis would make it more appealing to service providers, as improved functioning is usually related to reduced costs, and service users may find this therapy more attractive if it is helping them move toward a valued goal.

Cognitive remediation therapies differ from each other in the way they are provided (eg, in groups, individually, on a computer, or on paper). Previous meta-analyses either found no outcome heterogeneity or when there was heterogeneity the components of the therapies did not account for this variation. The Futures paper therefore suggested that differences between therapies were not important. But since then, data have emerged that suggest that some ingredients are important. This is also vital for implementation and providing the best outcomes to our patients. In a landmark study, Bowie et al^9^ produced a consensus on 4 key ingredients that should be included to call the treatment a cognitive remediation program. These field leaders were specifically concerned with improved functioning, reductions in disability and evidence that an ingredient of therapy helped this process. They are the practice of cognitive exercises, attention to the development of cognitive strategies, an active trained therapist, and procedures to facilitate transfer of cognitive gains to everyday functioning.

***Cognitive exercises*** are part of every cognitive remediation program, and the consensus was that multiple repetitions are needed, and that intensive practice was preferred although there was no agreement on the specific dose. More recent studies have shown that benefit increases with more sessions^49,50^ In addition, there was a recommendation that the exercises should gradually increase in difficulty, so they challenge but do not overwhelm the trainee. This is a process called scaffolded performance, so the exercise is within the competence of the individual but still stretches their ability.

Cognitive remediation treatment includes opportunities for participants to identify, develop, and monitor ***strategies*** for solving problems during cognitive training tasks. We know that individuals with a diagnosis of schizophrenia often use rigid and inefficient problem-solving strategies.^51^ The development, monitoring, and knowledge about potential strategies is part of metacognition and we will argue later that metacognition is essential for transferring gains in skills to the real world.

The ***therapist*** should be trained, and this training reported in cognitive remediation studies together with a formulation of the cognitive problem, link of treatment to the individual’s goals and progress, identifying barriers, and adjusting those goals. It was suggested that clinician praise should focus more on the process of training (eg, staying engaged with the task, attempting new strategies), rather than on overall performance to avoid negative attributions associated with perceived or actual low performance. The therapist is not passive but has an active role to focus attention on issues of importance, the development of strategic thinking, and the transfer of gains to the real world. We know that a cognitive remediation therapist can enhance the specific effects of therapy as well as having nonspecific but important effects. For instance, they can improve therapeutic alliance thus affecting engagement^52^ and improve strategy use.^53^ They are also valued by the participant^48,54,55^ and that affects perceived treatment usefulness^56^ and may also improve engagement in other treatments.^57^ The Futures paper discussed these therapist effects as specific and nonspecific but did not specify what they might be or their potential to influence outcomes. As a comparison, the specific elements of CBTp include guided discovery and a focus on cognitions, whereas the more general, nonspecific elements might consist of setting a session agenda, collaboration, and being responsive to feedback^58^ and fostering good therapeutic alliance.^59^ For cognitive remediation, specific contributions could include encouraging the development of strategic thinking, and the nonspecific elements could be positive feedback and engagement in activities. It is likely that for some individuals or for some therapies, the specific effects may have limited effects, but these may be counterbalanced by strong nonspecific effects. These therapist skills are important competencies and need further evaluation and incorporation into training.

The ***transfer of cognitive gains into the real world*** is part of the definition of cognitive remediation. Providing cognitive remediation with specific psychosocial rehabilitation programs will enhance functioning although functional improvements have been found without this formal approach. For transfer to be enhanced, we need realistic cognitive and functional goals and expectations, and that is part of the formulation approach. Embedding real-world examples or role-play of how cognition and metacognition can transfer into the community can help.^60,61^ Whatever the techniques for transfer, they are part of the program, especially the role of cognitive skills and strategies learnt during the treatment.

A recent meta-analysis tested 3 therapy ingredients as cognitive exercises are included in all programs. Each element was important for cognition and functioning benefits and including all ingredients produced significantly higher benefits for structured development of cognitive strategies (cognition: χ21, 9.34; *P* = .002; functioning: χ21, 8.12; *P* = .004), active and trained therapist (cognition: χ21, 4.14; *P* = .04; functioning: χ21, 4.26; *P* = .04), and integration with psychosocial rehabilitation (cognition: χ21, 5.66; functioning: χ21, 12.08) and when all 4 ingredients were present (global cognition: χ21, 5.66; *P* = .02; global functioning: χ21, 12.08; *P* < .001).^18^ In a further meta-analysis treatment acceptability was improved when all 4 elements were present.^20^ Active therapists were defined as a trained person in touch with the participant in a therapeutic way,^18^ so this removes therapies where they were facilitators who looked after the computer, turning it on and ensuring that there was adequate access. What is still unclear is how much therapist time is needed, and this was tested in a 4-arm study comparing treatment-as-usual with cognitive remediation in one-to-one, group, and independent (with limited therapist access) formats. There was a significant benefit of both group and one-to-one but little effect of the independent approach. However, there was little difference between the group and one-to-one methods,^49^ so we still do not know the extent of the involvement, only that, it is better to have more than the half an hour a week that was offered to the independent group.

**Fig. 1.**
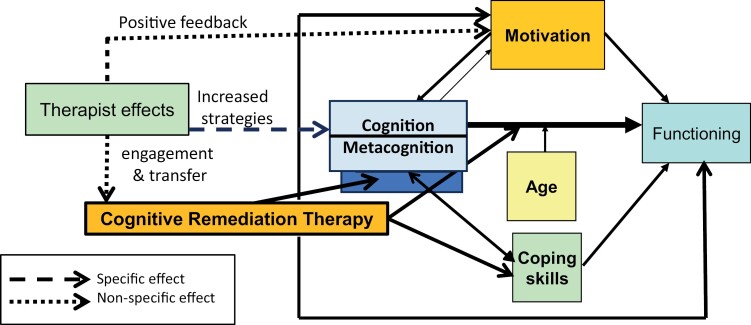
Potential therapist effects on aspects of CR. Adapted from Wykes and Spaulding^1^.

We have demonstrated that there is a difference between therapies with different labels and although 4 components do provide benefit there may be more, or more nuanced, descriptions of the current elements that will be important. It is unlikely that the simple model of remediation of cognition will have a direct effect on functional outcome, so the Futures paper produced a model including evidence-based variables. We have added to that model with the potential effects of therapists in figure 1 that might be investigated for the causal effects. This would support implementation by making it clearer what therapists should concentrate on in their interactions to gain the most treatment benefit. But we also need to consider whether there are further factors that can uncover the mechanisms of cognitive remediation that might allow a more tailored approach.

## Mechanisms, Moderators, and Mediators of Cognitive Remediation Effects

The Futures paper spent some considerable time considering the mechanisms of cognitive remediation with discussions of neural plasticity, neurophysiological reorganization, and psychological models. To those we can now add genetic contributions such as the alleles of COMT that interact with the other models. Much has been discussed about these models, but empirical evidence is not at all clear, and more importantly, these models do not seem to be useful in discerning the barriers to implementation. These theoretical models have not had much effect as there is a lot of similarity of treatment procedures and they have also had little effect on the narrative of treatment provision within services. Taking CBTp again as an example, it was implemented into treatment guidance before specific models were explicated. Since its inclusion, models of CBTp have been refined and used to highlight formulation and homework as important in realizing benefits.^62^ We have therefore stepped back and considered the usefulness of any CR model and considered individual factors that might affect benefit.

This means concentrating on mediators and moderators so that we can provide advice to health care services on whether this treatment should be provided to everyone or a select group, and how the most benefit can be achieved. The difficulty is that our results demonstrate how a factor, such as high levels of negative symptoms, reduce the benefits of cognitive remediation,^63^ but that does not mean that those people gain nothing. It may just mean that they need more time or an adjustment to the transfer of cognitive benefits to everyday life to achieve a similar benefit. So, moderators or mediators really provide us with a further challenge of how to reduce their effects by refining treatment techniques or tailoring treatment. We know that those with higher levels of community functioning do not seem have a long-term benefit from adding cognitive remediation to supported employment.^42^ Targeting those who would achieve benefit should reduce treatment costs while increasing overall supported employment outcomes.

Most models have assumed one important point—that the effect of cognitive remediation is primarily driven through boosting cognition. After all, as the Futures paper assumed, cognitive remediation does “*what it says on the tin*.” Despite correlations between improved cognition and the goal of functioning there have been few studies that have tested enhanced cognition as a mediator of functional improvement. Of those mediational analyses, most find only partial mediation.^64–71^ There are several reasons for a lack of a clear effect. Cognition may need to be improved above a certain threshold to translate into community functioning, or mundanely, it could be attributable to variation in cognition and functioning measures. But this lack of a defined effect may also be explained by moderators and some of these (eg, negative symptoms) were tested on data from a large cognitive remediation trial in a moderated mediation model that included therapy dose rather than just therapy exposure making it a more sophisticated analysis. The 2 moderators were total symptom severity on the link between therapy dose and cognition improvement, and negative symptoms on the translation of cognitive improvements to functioning. Cognition improvement was positively correlated with functional outcome, as has been found in other studies, but it was not a mediator. Symptoms did not moderate the effects of treatment on cognition suggesting that most people, irrespective of symptom severity, can benefit. However, cognitive improvement was only translated into functional benefits for those with low levels of negative symptoms.^63^ This indicates that we need some flexibility in the provision of cognitive remediation for those with higher negative symptoms, especially as the goal is to improve functioning. These individuals may need to have more sessions or be provided with more support to practice skills in the community or some other adaptation to achieve enough benefit for the service to be cost-effective.

Other treatment moderators have been advanced including participants’ age, education, and IQ but also biological, clinical, and illness-related features. The consensus from a recent systematic review is that there is poor replication of moderators across studies, and this has led to a long list of potential candidates.^72–74^

One aspect of cognition not yet interrogated is metacognition. This is divided into 2 parts—metacognitive knowledge and metacognitive regulation. Metacognitive knowledge is the presence and acquisition of strategies that can increase performance during cognitively demanding situations, as well as what we know about cognition and what can affect it (eg, performance anxiety). Metacognitive regulation is how strategies are planned, used, monitored, and adjusted based on feedback. We think metacognition is vital in transferring knowledge learnt within the cognitive remediation program to everyday life. We know that it is important in work outcomes for people with first episode psychosis^75^ and partially mediates the link between cognition and functional capacity, and fully mediates the relationship between functional capacity and functioning.^76^ It is also related to the experience of hallucinations.^77^ One cognitive remediation program, CIRCuiTS^TM^ has already been shown to increase metacognition.^78^ A definitive role within cognitive remediation has not yet been discovered and requires metacognitive measures to be used in trials—a further recommendation of this paper. The recognition of its importance may help us again to implement therapy effectively to produce the most benefit.

## An Updated But Not Simple Model of Cognitive Remediation Effects

**Fig. 2.**
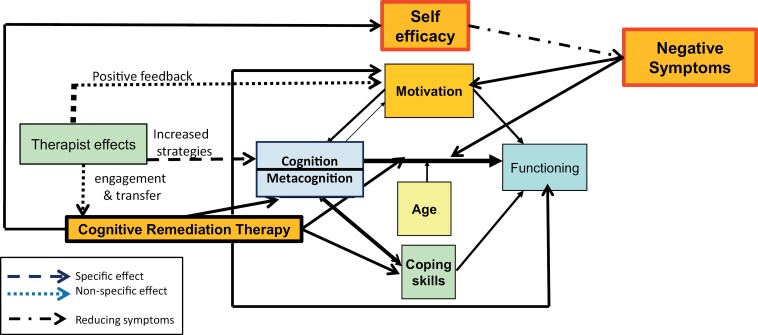
Mechanisms of cognitive remediation (so far). Adapted from Wykes and Spaulding^1^ and Tinch-Taylor et al.^63^

We have tried to incorporate the current evidence into the model shown in figure 2 using information from recent studies. It builds on Wykes and Spaulding model but considers the new information on specific and nonspecific effects. As the reader will see it is complex and the specific effects for some individuals may be weak and therefore produce a small effect on cognition but the nonspecific effects, related to the therapist and other environmental features, may be strong and therefore have a large effect on functioning. Small effects are not necessarily worthless as they may be the secret sauce for boosting skills useful in the community. Further work can examine the degree to which small proximal effects on cognition are nonetheless clinically significant and functionally important, particularly given commonly observed functional improvements that manifest later than cognitive changes.^79^ Together with the yet mostly unmeasured metacognition effects, this may explain the lack of complete mediation through the sole measurement of cognitive performance.

## Building on the Ingredients

Improvements in functioning also need extra support provided by opportunities to practice skills in the community such as adding cognitive remediation to work rehabilitation. But supported employment may be beyond a service’s resource or, more importantly, work may not be an individual’s main goal.^49^ Often individuals want more independence, gain living skills, an improvement in social relationships or to be put on the path to a career through education. To provide a more tailored intervention, we also need to provide formal rehabilitation programs and opportunities to practice skills in more ecologically sound environments. This is designated as the background effects in the model. Discussion groups or “bridging groups” are offered by some clinics^80^ that provide opportunities for understanding how specific skills learnt on simulation computer tasks might be used in everyday life. These depend on attendees suggesting ways they might integrate skills or, more powerfully, be tailored to a formulation agreed at the beginning of therapy. Another potential way is to integrate tasks into the software that allow a small step toward practising skills^81^ or to integrate in vivo practice in the clinic. Bowie et al worked with clinicians, community representatives (eg, employers), and those with lived experience to create props and materials for social, recreational, and vocational role plays that are paired with cognitive training tasks. This approach allows for the immediate practice, in a real-world simulation, of the cognitive skills and strategies developed in the same session.^61^ Another burgeoning area is “in vivo” training using virtual reality. This has the benefit of highly controlled opportunities that can be scaled in difficulty, unlike the sporadically available community opportunities. Software is available that can measure community functional capacity that is feasible and acceptable and might be used together with coaching to aid skill translation.^82^ Virtual reality is also flexible and could offer structured opportunities to practice specific valued skills and therefore a more personalized rehabilitation approach, such as job interview training.^83^

We also recommend adding cognitive remediation to other treatments as evidence suggests increased effects, eg, better community and work functioning,^84^ fewer sessions of CBTp if CR is provided first,^85^ and longer programs showing improved functioning at 5 years^86–88^

## Is Cognitive Remediation Just for Those With Schizophrenia?

Although most data have been collected for schizophrenia, there are parallel studies for those with neurological disorders,^89–91^ and depression.^92,93^ There is also an increased recognition that cognitive difficulties are significant and limit recovery in people with bipolar disorder^94,95^ and are a relevant illness dimension for a third of people with this diagnosis. Initial studies of euthymic bipolar disorder suggest acceptability, feasibility, and indicate efficacy.^96^ Models and delivery modality for this group resemble closely those used in people with a diagnosis of schizophrenia with small adaptations for clinical features that affect cognition (eg, impulsivity, poor monitoring).^96–98^ There are also studies in addictions^99,100^ and eating disorders^101,102^ that show promise.

Not only has cognitive remediation been used in different disorders, it has also been spread to different countries with the latest meta-analysis in schizophrenia^18^ considering 130 randomized controlled trials with 57 in Europe, 38 in the United States, 22 in Asia, 4 in Canada, 4 in Middle East countries, 3 in Australia, and 2 in Brazil. While this shows a skewed distribution of CR studies to western countries there is emerging evidence from low-income countries including studies in Sub-Saharan Africa, Togo and Benin, India, and Iran.^103–105^ These studies often use paper and pencil versions,^103^ demonstrating that this therapy can be translated and culturally adapted across all continents.

## Clinical Implementation

Clinical implementation begins with clinical health professionals accepting the importance of cognitive difficulties in the process of recovery. People with lived experience understand they have some problems, and we have data on acceptability and benefits of cognitive remediation. But this information either is not known to clinicians or is not part of their priorities. We need to consider how to overcome this barrier which is clearly possible as they have shown in France and Finland where cognitive remediation is available.^106,107^

### Measuring Benefit

We need information about the recovery benefits and costs as both have been identified as barriers and facilitators for implementation.^108^ As cognitive remediation has the goal of improving functioning, we need to consider what outcome is appropriate for our trials and is valued by both service users and service providers and most importantly is useful for clinical implementation. We have already discussed the problem that a general measure is unlikely to be appropriate for all users, as recovery trajectories and ambitions change over time and are different at the early compared to later stages. In line with service user and provider preferences, we favor measures of personal recovery (the Goal Attainment Scale^109^; Questionnaire for the Process of Recovery^110^) as the primary outcome. This comes with advantages, particularly their value to users and providers, but also has the downside that these outcomes are not those generally measured in trials. The adoption of a more personalized outcome might help communicate the value of cognitive remediation and especially how it can deal with the different recovery goals.

### Training

Knowledge, competencies, and skills around cognitive assessment and difficulties in people with severe mental health difficulties are limited. They are rarely part of training curricula and, even if present are unlikely to provide cognitive assessment skills. This lack of knowledge can lead to a reframing of cognitive difficulties as resulting from more understood problems, eg, experiencing disorganized behavior because of thought disorder as opposed to resulting from executive difficulties. To enhance training several programs have been produced. One online evaluated program demonstrates learned competencies, but it requires manager buy-in to ensure the program is completed.^111^ A recent national platform for training and credentialling cognitive remediation clinicians in Canada is being implemented.^112^ These approaches suggest the potential of training enough clinicians for large-scale rollout. However, a clear focus on an agreed set of competencies would make training development easier, in the same way that CBTp has clear templates and standards.

### Formulation

We have produced a model including factors that are important for cognitive remediation benefit. These should form the basis of a formulation on barriers and facilitators for therapy benefit. We know there is cognitive heterogeneity^5^ so we need to assess a wide range of cognitive domains to identify strengths and difficulties. With the introduction of a potentially important factor, metacognition, clinical academics need to suggest some short measures to inform clinical practice so therapists can build the supports into the program and measure the benefits. Symptoms, especially negative symptoms, might interfere with the translation of cognitive benefits to real-world activities. We therefore need to consider lengthening the therapy and provide more opportunities for transfer to those clients. Motivation is allied with valuing therapy benefits and so that means discovering personal goals that can be linked to cognition. With all these, and potentially more, aspects affecting outcomes it is important that formulation models are routinely considered when delivering cognitive remediation so that the therapy is meaningfully contextualized in individuals’ unique sets of strengths and difficulties.

## Future Research

Given that we know cognitive remediation works (even if the effects are statistically modest), that it is cost-effective, and that we can train therapists, then future research should pay attention to specific and nonspecific effects of therapy and how benefits can be measured. This would allow us to build models of factors known to be influential in achieving therapy benefit and would advance our understanding of what to assess before and during therapy. In the Futures paper, we advocated pooling data, and, with the help of NIMH, the DOCTRS database was produced that allowed testing of moderators and mediators.^70,71,113^ This is clearly the best approach to open science but is much more time consuming than the Futures paper envisaged. Without some agreement on measures, the task of comparing individual-level data will continue to be tedious. There was also a suggestion that we would begin to measure the costs and cost-effectiveness of cognitive remediation and that has materialized. This should remain on our agenda for the next few months, not years as it is relatively easy to work out how much each therapy costs to provide, including the training and therapist’s costs.

In the Futures paper, we also produced a table of issues to consider in our research, entitled “An agenda for improving what works in cognitive remediation.” We have re-jigged this to remove what we think has been achieved and what is left to consider clinicians, service providers, clients, and clinical academics. Table 1 considers the issues for specific research and wider implementation and acts as an agenda for improving our understanding of cognitive remediation and what a clinician needs to consider in order to provide this therapy in services. In this list, and this paper, we only considered non-biological issues, but it is likely that these factors too will affect our understanding of the mechanisms and tailoring of treatment.

**Table 1.** An Agenda for Improving Treatment Through Research and for Delivering Cognitive Remediation into Services

Category	Issue	Recommendations for Understanding How Therapy Works	Recommendations for Supporting Wider Use and Clinical Benefits
Participant characteristics	Cognitive strengths and difficulties	- Evaluate the relevance of cognitive reserve	- Identify specific and general strengths and difficulties (see outcomes below)
	Noncognitive strengths and difficulties	- Symptoms interaction with therapy components	- Personal background and support - Medication and other aspect affecting the person (eg, sleep poorly)
	Participant approach to therapy	- Motivation interaction with therapy components	- Measure likely therapy engagement (estimate of worth, understanding of) - Personal goals identified
Therapy characteristics	Key components	- Identifying other beneficial ingredients eg, session intensity and length - How ingredients interact with characteristics of the trainee - Testing therapies against each other - Testing whether adding in vivo or VR practice to enhance transfer of skills to the community	- Choosing therapy containing the 4 key ingredients currently identified (cognitive exercise, teaching strategies, an active therapist, transfer process to everyday life) - Identify personal recovery goals - Detecting which rehabilitation efforts added to cognitive rehabilitation enhance benefit
	Therapist	- Evaluate how alliance and therapist’s skills and training contribute to outcomes - Define and measure specific and nonspecific effects (eg, strategy teaching, positive feedback)	- Define therapist basic skills - Measure fidelity to treatment - Measure clinical alliance with therapist - Promote learning about own’s cognition
Outcomes wider adoption	Specific vs General cognitive measures	- Therapy effect on basic processes, eg, reward learning - Therapy effect on biological outcomes, eg, brain functionality - Measuring metacognition to inform therapist actions and refine cognitive remediation mechanisms - How much cognitive change is enough to change functioning?	- General cognitive improvement - Investigate patterns of performance within and across cognitive domains
	Translational outcomes	- Therapy process measures (eg, strategy learning, improvement on tasks)	- Personal goals - Self-efficacy or self-esteem Motivation
	Initial acceptance		- Health care professionals know and accept cognitive health is important - Cognitive health is a priority for all patients
	Health economic factors		- Measure costs of therapy - Measure cost-effectiveness and/or cost utility to understand potential future improvements
	Resources	- Evaluate how resources contribute to different outcomes	- Clarify all resources needed (eg, computer, internet) - Clinical supervision and grade or profession of the therapist - Licenses for computerized therapy - Therapist training - Training on cognitive difficulties
	Information to service providers, clinicians, and potential clients	- Evaluate how health care system policies influence therapy delivery (eg, individual therapy session number is limited)	- Adoption by professional organizations - Publicly available information on benefits - Adoption by advocacy groups - Clinical guidelines

## Some Final Thoughts

Cognitive remediation is not provided in a vacuum, so we owe it to our clients to understand their environment and the opportunities it offers for practising skills and allowing their ambitions to be achieved. Most people with long-term conditions do not have the same financial or social opportunities available to others. Economic circumstances affect whether there are jobs available or whether the skills necessary are within an individual’s capability or interest. All these factors affect the agreed formulation that starts therapy. Goals need to be achievable and so a therapist must understand the psychosocial demands and barriers as well as opportunities for any therapy to provide benefit. Cognitive remediation is therefore not mechanical—we do not simply assess cognition then provide computerized therapy. It is a negotiation on the strengths and weaknesses that might help or impede ambition to increase the acceptability and meaningfulness of the therapy to our clients.

In conclusion, we have a process for therapy, we have shown benefits and know that training therapists is possible for large-scale rollout. But (and it is still, nearly a decade after the original Futures paper, so a big “but”), there are few public relations or campaigning groups to advertise this therapy to clinicians and particularly to those holding the purse strings. For this psychological therapy to be implemented more widely we need to have a group who will provide that publicity. We need advocacy groups, particularly patients and carers, to provide support for its inclusion in treatment regimens and so reduce the waiting times for an effective therapy. As it begins to be adopted into services, we will need a group of specialists to ensure that the quality of treatment reaches a required standard so that cognitive remediation can continue to provide the most benefit.
